# Development and Refinement of a Chatbot for Birthing Individuals and Newborn Caregivers: Mixed Methods Study

**DOI:** 10.2196/56807

**Published:** 2024-11-14

**Authors:** Jessica Nathalie Rivera Rivera, Katarina E AuBuchon, Marjanna Smith, Claire Starling, Karen G Ganacias, Aimee Danielson, Loral Patchen, Janine A Rethy, H Joseph Blumenthal, Angela D Thomas, Hannah Arem

**Affiliations:** 1 Healthcare Delivery Research Network MedStar Health Research Institute Washington, DC United States; 2 Lombardi Comprehensive Cancer Center Georgetown University Washington, DC United States; 3 Department of Pediatrics MedStar Georgetown University Hospital Washington, DC United States; 4 Department of Psychiatry Georgetown University School of Medicine Washington, DC United States; 5 Obstetrics and Gynecology MedStar Georgetown University Hospital Washington, DC United States; 6 Obstetrics and Gynecology MedStar Washington Hospital Center Washington, DC United States; 7 Center for Biostatistics, Informatics and Data Science MedStar Health Research Institute Washington, DC United States; 8 Department of Oncology Georgetown University Washington, DC United States

**Keywords:** postpartum care, newborn care, health education, chatbot, mHealth, mobile health, feedback, health equity

## Abstract

**Background:**

The 42 days after delivery (“fourth trimester”) are a high-risk period for birthing individuals and newborns, especially those who are racially and ethnically marginalized due to structural racism.

**Objective:**

To fill a gap in the critical “fourth trimester,” we developed 2 ruled-based chatbots—one for birthing individuals and one for newborn caregivers—that provided trusted information about postbirth warning signs and newborn care and connected patients with health care providers.

**Methods:**

A total of 4370 individuals received the newborn chatbot outreach between September 1, 2022, and December 31, 2023, and 3497 individuals received the postpartum chatbot outreach between November 16, 2022, and December 31, 2023. We conducted surveys and interviews in English and Spanish to understand the acceptability and usability of the chatbot and identify areas for improvement. We sampled from hospital discharge lists that distributed the chatbot, stratified by prenatal care location, age, type of insurance, and racial and ethnic group. We analyzed quantitative results using descriptive analyses in SPSS (IBM Corp) and qualitative results using deductive coding in Dedoose (SocioCultural Research Consultants).

**Results:**

Overall, 2748 (63%) individuals opened the newborn chatbot messaging, and 2244 (64%) individuals opened the postpartum chatbot messaging. A total of 100 patients engaged with the chatbot and provided survey feedback; of those, 40% (n=40) identified as Black, 27% (n=27) identified as Hispanic/Latina, and 18% (n=18) completed the survey in Spanish. Payer distribution was 55% (n=55) for individuals with public insurance, 39% (n=39) for those with commercial insurance, and 2% (n=2) for uninsured individuals. The majority of surveyed participants indicated that chatbot messaging was timely and easy to use (n=80, 80%) and found the reminders to schedule the newborn visit (n=59, 59%) and postpartum visit (n=66, 66%) useful. Across 23 interviews (n=14, 61% Black; n=4, 17% Hispanic/Latina; n=2, 9% in Spanish; n=11, 48% public insurance), 78% (n=18) of interviewees engaged with the chatbot. Interviewees provided positive feedback on usability and content and recommendations for improving the outreach messages.

**Conclusions:**

Chatbots are a promising strategy to reach birthing individuals and newborn caregivers with information about postpartum recovery and newborn care, but intentional outreach and engagement strategies are needed to optimize interaction. Future work should measure the chatbot’s impact on health outcomes and reduce disparities.

## Introduction

Maternal mortality rates in the United States are increasing; in 2018, the maternal mortality rate was 17.4 per 100,000 live births, nearly doubling by 2021 to 32.9 per 100,000 live births [1]. Furthermore, there are stark inequities for Black women, who in 2021 suffered 69.9 deaths per 100,000 live births compared to 26.6 for non-Hispanic White and 28 for Hispanic women [1]. While about half of all deliveries in Washington, DC are among non-Hispanic Black women, data from 2014 to 2018 indicate that 92% of all maternal deaths occurred among non-Hispanic Black women [2]. Infant deaths in the United States are also unacceptably high, at a rate of 5.4 deaths per 1000 births, but even higher in Washington, DC, at 6.8 per 1000 births [3,4]. Non-Hispanic Black women and infants bear the primary burden of these mortality inequities. Racially and ethnically minoritized individuals experiencing the highest risk of maternal and infant mortality may have lower access to care due to structural discrimination [5,6].

In the “fourth trimester” (first 42 days after birth), there are significant caregiving demands, physical recovery from childbirth, and emotional challenges [7]. Despite the high-risk nature of this period, birthing individuals in the United States experience little support for postpartum health, and some have challenges accessing a visit 6 weeks after delivery [7]. Missing postpartum care has devastating health consequences: 65% of pregnancy-related deaths occur in the first year after delivery, 35% of which occur in the first 42 days after delivery [8]. An estimated 60% to 84% of pregnancy-related deaths are preventable [8,9], and insufficient knowledge regarding warning signs of complications is identified as the most common factor contributing to pregnancy-related death [9]. In relation to infant mortality, 64% of infant deaths occur in the first 27 days, and about 14% of infant deaths are attributed to sudden infant death syndrome and unintentional injuries [3], some of which could be prevented with education [10]. Increased awareness of postnatal warning signs through universal education may empower birthing individuals to call their health care provider and to know when to seek immediate care [11,12]. For newborns, prompt follow-up care with a pediatrician and understanding warning signs to seek care may also decrease adverse events [13].

Digital technologies may help provide timely and trusted health information to patient populations [14] including birthing individuals [15]. Digital technology is often promised as an opportunity to reduce some educational burden on providers and increase patient knowledge [16]. Chatbots are one type of digital tool for delivering health information on a variety of topics, which allow for patient interactivity through mimicked conversation [16,17]. Chatbots are defined as any software programs simulating human conversation, which vary from rule-based, decision tree-style menus of options to advanced technologies relying on artificial intelligence (AI) [18,19]. Overall, chatbots have demonstrated a high acceptability rate among birthing individuals and newborn caregivers [20-22] and are effective at delivering interventions targeting birthing individuals’ depression [23] and anxiety [24], as well as helping parents identify newborn developmental delays [25]. While these studies provide promise for health information delivery via chatbot, there is a need to further understand how chatbot outreach is received in real-world settings, particularly among historically marginalized birthing individuals.

The goal of this study is to describe the development and refinement of chatbot outreach and content for postpartum individuals and newborn caregivers delivered as part of the standard of care. Postimplementation, we surveyed and interviewed socioeconomically, racially, and ethnically diverse patients who received chatbot messaging to improve the chatbot outreach and content.

## Methods

### Chatbot Development

**Figure 1:**
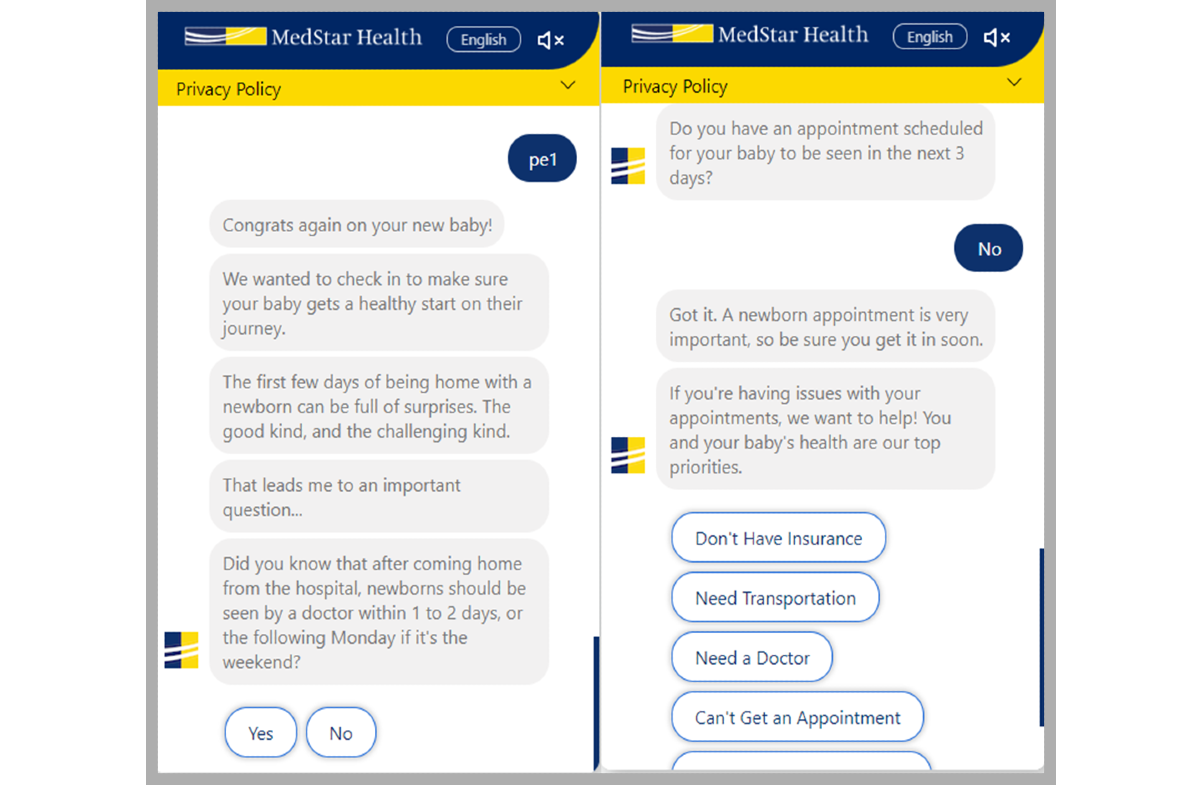
Newborn chatbot examples.

**Figure 2:**
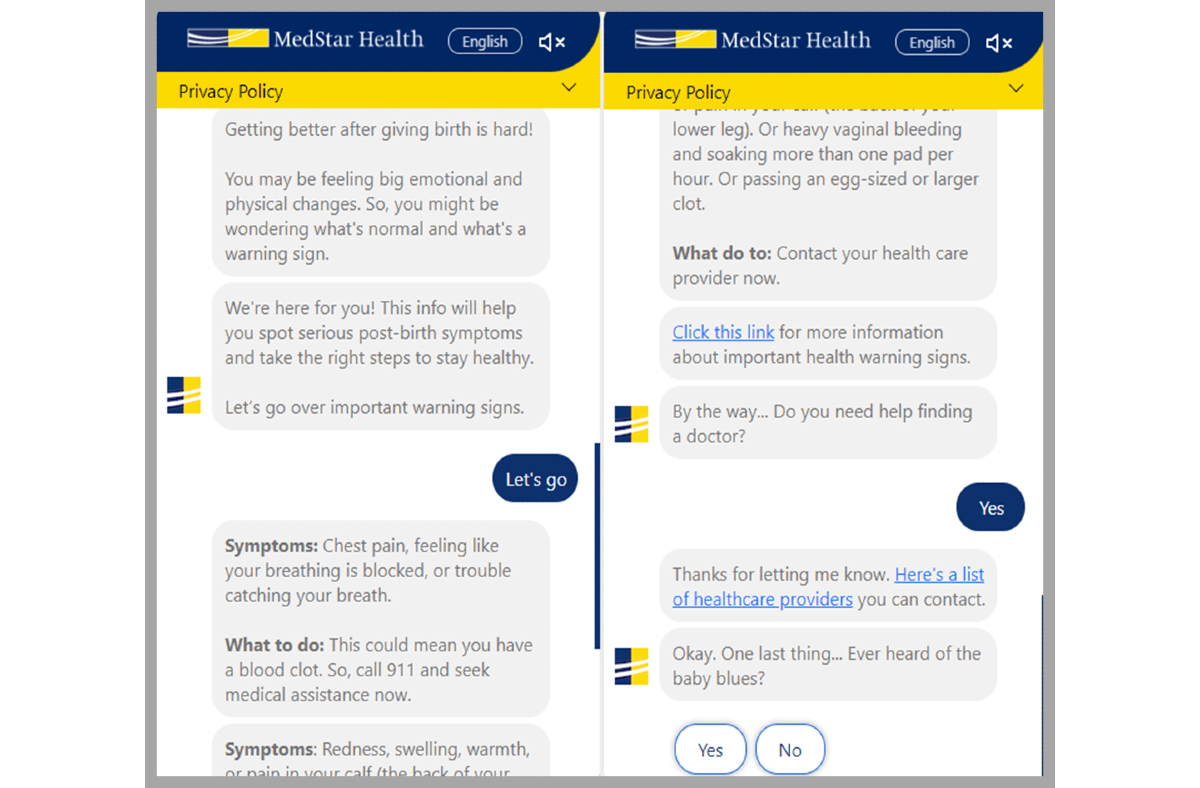
Postpartum chatbot examples.

We followed a person-centered approach to develop and refine a chatbot for postpartum individuals and newborn caregivers, with an emphasis on incorporating the perspectives and unique needs of our socioeconomically and racially diverse patient population [26]. The chatbot content was developed by a multidisciplinary team of experts in obstetrics, pediatrics, social work, psychiatry, mobile health, and health equity. The chatbot is intended to promote connection to care teams, provide education about warning signs, and deliver other postpartum and newborn information and resources (Figures 1 and 2). Two separate messaging streams (postpartum and newborn) were created because (1) newborns and postpartum patients are not always discharged together, and (2) some individuals may only need postpartum or just newborn content (eg, if there is a stillbirth, adoption, etc). We obtained a HIPAA (Health Insurance Portability and Accountability Act) waiver to use electronic health record (EHR) data to examine whether there was equitable chatbot engagement (defined as opening the chatbot link from the outreach message) by various patient characteristics.

### Survey and Interview Recruitment

We recruited English- and Spanish-speaking birthing individuals and newborn caregivers from a diverse, mid-Atlantic urban hospital. We generated a list of potential participants from the EHR, purposively selected by age, racial or ethnic group, insurance type, and prenatal care location. Individuals were contacted via email and phone to invite them to complete either (1) a brief web-based survey that was conducted via REDCap (Research Electronic Data Capture; Vanderbilt University) or (2) a semistructured interview that was conducted by phone or video call using Teams (Microsoft Corp).

For web-based surveys, we invited a distinct list of respondents balanced across those who did and did not engage with the chatbot. Of the 410 individuals contacted for the survey completion, 104 (25%) individuals completed the informed consent, and 100 (24%) individuals completed the survey between May 15, 2023, and August 8, 2023. For interviews, we invited individuals who opened the chatbot. Of the 110 individuals contacted, 28 (25%) individuals agreed to participate, and 23 (21%) individuals completed a semistructured interview between March 13, 2023, and June 1, 2023; the remainder missed the scheduled interview time and were not interested in rescheduling.

### Ethical Considerations

The study protocol was approved by the institutional review board from MedStar Health Research Institute (IRB #5741). All participants provided informed consent, and data were anonymized for analysis. Individuals were compensated US $10 and US $50 for their participation in the survey and interview, respectively.

### Demographic Data Collection

Demographic data related to the study was obtained by directly querying the integrated enterprise EHR system’s database to describe patients’ general chatbot engagement. We included race (Back or African American, White, other, or unknown), age, and insurance type (private or commercial, Medicare or Medicaid, other, uninsured, or unknown) of the birthing individual. For the survey and interviews, we collected self-reported sociodemographic characteristics of gender identity (woman, man, nonbinary, transgender, or other), race (American Indian or Alaska Native, Asian, Back or African American, Native Hawaiian or Other Pacific Island, White, or Other), and ethnicity (Hispanic vs non-Hispanic). For the survey, participants reported their age, sexual orientation, highest level of education completed, English literacy level, employment status, living arrangement, and type of health insurance. Age and insurance type were obtained from the medical records of interview participants. Participants of both the survey and interview also reported the number of times they gave birth, the number of children they cared for, prenatal and postpartum care locations, newborn care, and if they had support at home recovering from childbirth and with newborn care.

### Survey Measures

Survey questions about the chatbot experience covered three domains: outreach strategies, usefulness of content, and usability. Outreach questions included, “How often did you open the links provided in the emails and SMS text messages?” and “Would you say the timing of the messages was: too frequent, just right, or not enough?” (response options: never, rarely, sometimes, often, or always). Regarding content, participants rated their level of agreement to a set of statements regarding the usefulness of the topics included in the chatbot experience (strongly disagree=1 to strongly agree=5). Regarding usability, participants rated their level of agreement with the statement “The chatbot was easy to use” (strongly disagree=1 to strongly agree=5) [27]. Participants also rated their level of agreement for the following statement “I would recommend the chatbot to other people” (strongly disagree=1 to strongly agree=5).

### Interview Process

We approached the interviews from a pragmatic qualitative framework [28], which emphasizes discovering problems, information gaps, and real-world solutions to the problem. Thus, we developed a standardized semistructured interview guide with a focus on the pragmatic understanding of postpartum and newborn concerns, how to improve chatbot engagement, and increasing engagement with chatbot content (Table 1). The interview guide was tailored by engagement; for example, if a participant did not remember receiving the outreach messages, they were asked to describe factors that typically influenced whether they opened SMS text messages and emails from their health care provider. Prior to and during the interview, participants received and were shown screenshots of outreach emails and SMS text messages, the chatbot interface, and the lists of topics included in both chatbots.

**Table 1:** Interview guide sample questions.

Domains	Example questions
Health concerns	What were your biggest health concerns for you or your baby after discharge from the hospital?
Chatbot outreach strategies	Can you tell me your impressions of the SMS text messages and emails you received after your hospital discharge? What did you think of the timing when the messages arrived?
Chatbot content	What are your thoughts about the topics that we included in the birthing recovery or postpartum chatbot? What are your thoughts about the topics that we included in the newborn chatbot? What could we do to better support birthing individuals or caregivers in accessing these resources?
Chatbot usability	What aspects of the chatbot were most helpful? What aspects of the chatbot were least helpful? How hard was it to use the chatbot?

### Data Analysis

Audio recordings from the interviews were transcribed and reviewed for quality control using Otter.ai and coded using Dedoose (SocioCultural Research Consultants). Three research team members (JNRR, CS, and MS) met to create the initial codebook with operational definitions for each code. The three team members then coded two randomly selected interviews individually and finalized the codebook together. The team members then triple-coded 3 additional interviews to meet an interrater reliability of 80% with JNRR. The team members independently coded the remaining interviews, with regular meetings to discuss inconsistencies and coding questions. We selected representative quotes and demonstrated theme salience by summarizing whether the sentiment was reported across most, around half, or a limited number of participants. Given the small number of interviews and the research team’s interest in the depth of response rather than the quantity of responses, we did not present statistics on the number of individuals reporting a specific theme. Descriptive statistics were used to describe the quantitative data using SPSS Statistics (version 29; IBM Corp).

## Results

### Chatbot Strategy

The initial postpartum chatbot outreach message was sent the day after the birthing individual was discharged home from the hospital, and the first newborn chatbot message was sent to the newborn caregiver listed in the medical record the day after their newborn was discharged from the hospital. The postpartum chatbot outreach SMS text messages and emails were sent in the morning and the newborn messages were sent in the afternoon. In Table S1 in Multimedia Appendix 1, there is a full description of the original chatbot content by day of the outreach message. The content was rule-based and allowed for interaction using fixed logic; it did not permit open-ended questions from the user or responses from a care team. Both chatbots were sent approximately weekly during the first 42 days of posthospital discharge.

### Overall Chatbot Engagement

In regard to the postpartum chatbot, a total of 2244 (64%) birthing individuals opened the chatbot (Table 2) between November 16, 2022, and December 31, 2023. When evaluated by racial group, 62% (n=969) of the Black users, 78% (n=384) of the White users, and 62% (n=891) of those classified as Other opened the postpartum chatbot. In addition, the proportion of patients who opened that chatbot increased by age; for example, 55% (n=183) of birthing individuals 21 years old and younger opened the chatbot compared to 68% (n=429) of birthing individuals aged 37 and older. By insurance type, 69% (n=971) of birthing individuals with private or commercial insurance and 63% (n=631) with Medicaid or Medicare insurance opened the chatbot.

A total of 2748 (63%) caregivers opened the newborn chatbot between September 1, 2022, and December 31, 2023. By racial group, 61% (n=1256) of the Black caregivers, 81% (n=449) of the White caregivers, and 61% (n=593) of those classified as Other opened the newborn chatbot. In addition, 58% (n=246) of caregivers 21 years old and younger and 69% (n=516) of caregivers aged 37 and older opened the chatbot. By insurance type, 71% (n=1185) of caregivers with private or commercial insurance and 61% (n=799) with Medicaid or Medicare insurance opened the newborn chatbot.

**Table 2:** Demographic characteristics of patients who received and opened the postpartum and newborn chatbot.

			Postpartum chatbot							Newborn chatbot				
			Total received (n=3497)		Total opened (n=2244)		Percentage opened (n=64.2)		Total received (n=4370)			Total opened (n=2748)		Percentage opened (62.9)
**Race**														
	Black	1566		969		61.9		2069			1256		60.7	
	Unknown	0		0		0		768			450		58.6	
	White	491		384		78.2		553			449		81.2	
	Other	1440		891		61.9		980			593		60.5	
**Age (in years)**														
	≤18	93		49		52.2		113			58		51.3	
	19-21	242		134		55.4		308			188		61	
	22-26	561		341		60.8		747			421		56.4	
	27-31	963		611		63.4		1199			743		62	
	32-36	1003		680		67.8		1260			822		65.2	
	37-41	543		364		67.0		640			445		69.5	
	>41	92		65		70.7		103			71		68.9	
**Insurance**														
	Private or commercial	1404		971		69.2		1675			1185		70.7	
	Medicare or Medicaid	1008		631		62.6		1304			799		61.3	
	Other	666		394		59.2		852			458		53.8	
	Uninsured	201		120		59.7		223			121		54.3	
	Unknown	218		128		58.7		316			185		58.5	

### Demographics for Survey and Interview Participants

Participants’ average age was 32 (SD 5.38) years old in the surveys, and 31 (SD 7.05) years old in the interviews (Table 3). A total of 40% (n=40) of survey participants and 61% (n=14) of interview participants identified as Black, and 27% (n=27) of survey participants and 17% (n=4) of interview participants identified as Hispanic. Over half of the Hispanic participants (18/27, 67% in the surveys and 2/4, 50%) in the interviews) identified Spanish as their preferred language. One-third of the participants in both the surveys and interviews indicated that this was their first time giving birth. About half of the participants in the survey (n=55, 55%) and interviews (n=11, 48%) had public insurance. Most of the survey and interview participants reported that they had a postpartum provider (n=78, 78% and n=22, 96%, respectively) and primary care provider (n=73, 73% and n=18, 78%, respectively) and that their newborns were receiving care outside of the integrated health care system (n=84, 84% and n=20, 87%, respectively).

**Table 3:** Participants’ demographic and clinical characteristics for survey and interviews.

Characteristics		Survey (n=100)	Interview (n=23)
**Age (years)**			
	Mean (SD)	32 (5.38)	31 (7.05)
	Range	19-42	19-43
**Sex, n (%)**			
	Female	99 (99)	23 (100)
	Male	1 (1)	0 (0)
**Sexual orientation, n (%)**			
	Straight	83 (83)	—^a^
	Other	11 (11)	—
	Prefer not to say	6 (6)	—
**Ethnicity, n (%)**			
	Hispanic	27 (27)	4 (17)
	Non-Hispanic	67 (67)	19 (83)
	Prefer not to say	6 (6)	0 (0)
**Race, n (%)**			
	Black only	40 (40)	14 (61)
	Black + another racial group	3 (3)	2 (9)
	White only	23 (23)	4 (17)
	Other only	24 (24)	3 (13)
	Prefer not to say	10 (10)	0 (0)
**Relationship status, n (%)**			
	Partnered or married	63 (63)	—
	Single or separated or divorced or widowed	29 (29)	—
	Other	3 (3)	—
	Prefer not to say	5 (5)	—
**Education level, n (%)**			
	< High school	9 (9)	—
	High school degree or equivalent	39 (39)	—
	≥College degree	49 (49)	—
	Prefer not to say	3 (3)	—
**Employment status, n (%)**			
	Employed	62 (62)	—
	Other	34 (34)	—
	Prefer not to say	4 (4)	—
**Living arrangement, n (%)**			
	Owner	30 (30)	—
	Renter	54 (54)	—
	Other	11 (11)	—
	Prefer not to say	5 (5)	—
**Times given birth**			
	Mean (SD)	2 (1.02)	2 (1.52)
	Range	1-5	1-7
**Number of children**			
	Mean (SD)	2 (0.89)	2 (1.48)
	Range	1-4	1-7
**Insurance type, n (%)^b^**			
	Private or commercial	39 (39)	12 (52)
	Medicare or Medicaid	55 (55)	11 (48)
	Other	3 (3)	0 (0)
	Uninsured	2 (2)	0 (0)
	Prefer not to say	6 (6)	0 (0)
**Prenatal location, n (%)**			
	Internal clinics	47 (47)	12 (52)
	External clinics	52 (52)	11 (48)
	Prefer not to say	1 (1)	0 (0)
	Connected to postpartum care	78 (78)	22 (96)
	Has a primary care provider	73 (73)	18 (78)
**Newborn care, n (%)**			
	Within an integrated health care system	14 (14)	3 (13)
	External to integrated health care system	84 (84)	20 (87)
	Prefer not to say	2 (2)	0 (0)
**Postpartum support, n (%)**			
	Yes or sometimes	87 (87)	20 (87)
	No	9 (9)	3 (13)
	Prefer not to say	4 (4)	0 (0)
**Newborn support, n (%)**			
	Yes or sometimes	89 (89)	20 (87)
	No	8 (8)	3 (13)
	Prefer not to say	3 (3)	0 (0)
**English literacy, n (%)**			
	Very well or well	83 (83)	—
	Not well or not at all	14 (14)	—
	Prefer not to say	3 (3)	—
**Preferred language, n (%)**			
	English	82 (82)	21 (91)
	Spanish	18 (18)	2 (9)

^a^Not applicable.

^b^Participants could select >1 option.

### Outreach Strategies: Satisfaction and Recommendations

Most survey participants (n=83, 83%) reported that they understood why they were receiving the outreach messages (Table 4). However, many interview participants reported being surprised when they received the messages, as they did not expect them (Table 5). Still, participants in the interviews found the outreach messages to be helpful. Participants recommended that the chatbot should be introduced both in prenatal care and by the recovery care team prior to discharge.

Interview participants reported that they opened the outreach messages because the messages came from the hospital where they delivered. Most participants in the survey (n=70, 70%) and interview preferred receiving messages via text because it was more personal, easier to notice, and involved fewer steps than email. However, during the interviews, it was noted that those who preferred emails did so because they could open the chatbot on their computer, which provided additional confirmation that the outreach messages were legitimate and not spam or scam.

More than half of the survey participants (n=56, 56%) indicated they opened the weekly chatbot link often or always. In the interviews, some participants reported that their decision to open each of the weekly chatbot experiences was based on the information or topic in the outreach message, as they only opened the chatbot experiences that were relevant to them. The interview participants also identified barriers to chatbot engagement including feeling overwhelmed with information, multiple household responsibilities, and confusing the chatbot with a survey.

Participants reported that the outreach messages were useful reminders of self-care and feeling supported by the hospital. Participants liked that the outreach messages were short, straightforward, and could be opened at any time, as opposed to a phone call which would be missed if they were not available. Participants also liked when the messages indicated what information would be covered in the chatbot experience. They recommended that the initial outreach should provide more information about why they are receiving the messages, and that all the messages should indicate what will be covered in each of the chatbot experiences. Furthermore, participants recommended making the messages more inviting, personal, and targeted. Some participants expressed that the chatbot’s wording should be appropriate to a diverse literacy level; for example, a few participants noted that the term “postpartum” could be confused with postpartum depression, and one participant suggested changing the term “chatbot” to something more friendly like “check-in messages.” One participant talked about a previous experience with stillbirth and suggested the postpartum chatbot would have been useful as she recovered.

**Table 4:** Survey feedback on outreach strategies and usability (n=100).

				Value, n (%)
**Outreach strategies**				
	**Confused with chatbot message**			
		Yes	7 (7)	
		Somewhat	10 (10)	
		No	83 (83)	
	**Preferred outreach strategy**			
		Email	30 (30)	
		SMS text messages	70 (70)	
	**Frequency of opening the chatbot**			
		Never or rarely	11 (11)	
		Sometimes	33 (33)	
		Often or always	56 (56)	
	**Timing of messages**			
		Too frequent	10 (10)	
		Just right	80 (80)	
		Not enough	10 (10)	
**Usability**				
	**Easy to use**			
		Strongly agree or agree	81 (81)	
		Neutral	16 (16)	
		Strongly disagree or disagree	3 (3)	
	**Would recommend the chatbot**			
		Strongly agree or agree	72 (72)	
		Neutral	22 (22)	
		Strongly disagree or disagree	2 (2)	
		Not applicable	4 (4)	

**Table 5:** Example of participants’ interview responses by themes.

Themes	Quotes
Outreach strategies and timing	“At the beginning, when I received the first message, I wasn’t sure what to do, if should read it or not, later I decided to read it and I found it to be good, because it was good information.” [Hispanic/Latina, Other, 38 years, female, 2 children] “In the past, I was a victim of identity theft. I’m very leery about getting messages from different places ... It was one time where I thought that it was a scam but when I saw it said [health care system], that was my indication that it was something real, something important, and informational.” [Non-Hispanic, Black, 35 years, female, 6 children] “So the text here is quick, easy, it’s telling you to the point, we know you’re busy, you got a lot going on ... And typically, that’s what people need these days ... There’s something about that quick.” [Non-Hispanic, Black, 40 years, female, 2 children] “It was really helpful to have both emails and text messages, just as a follow-up in case I miss one or the other. But I felt it was like perfect timing, because it checked up on me.” [Non-Hispanic, White, 31 years, female, 1 child] “So, I may have opted out a little early. Which I feel like even though I did opt out I wish there was like another option that you know, I can get back in it ... Because at one point in time, I wish I was receiving the messages again after I opted out.” [Non-Hispanic, Black, 25 years, female, 1 child]
Knowledge and information-seeking	“I just relied solely on my family, I didn’t really have resources to reach out to anything ... I didn’t really have health concerns for myself, but for my son, it was just more so how to care for a baby that just got out the NICU. I didn’t really have ... much information on things like that. So it was kind of like I had to learn by myself. And figure things I don’t know.” [Non-Hispanic, Black, 25 years, female, 1 child] “There’s some weird things that happened with the babies. There’s like, how do you care for their umbilical cord? Once that falls off ... what do you do with it? That was a question I definitely Googled. They have weird skin stuff. What is normal? What’s concerning?” [Non-Hispanic, White, 30 years, female, 2 children]
Chatbot content	“I learned how to take care of my son, I learned how to take care of myself, [I received them] just recently leaving the hospital, I learned a lot of stuff.” [Hispanic/Latina, Other, 29 years, female, 3 children] “I didn’t know anything about postpartum until I had my baby. So, really understand because it’s like postpartum depression, like what you’re talking about.” [Non-Hispanic, Black, 22 years, female, 2 children] “The one thing I can say I really enjoyed receiving messages about were the developmental milestones, because I always wanted to know if my baby was on track, being as he was premature baby ... And as a first-time parent ... It can be really overwhelming ... So being as though [health care system] was giving you information like vaccines, and you know, what to expect with these vaccines and things like that. I feel like that was very helpful...” [Non-Hispanic, Black, 25 years, female, 1 child] “I was glad that it was put together because usually that is the time that I go through the worst end of postpartum depression. But even though I didn’t have the effects of postpartum depression, like I usually do, this time around, I was just glad that it was something I could read to give, I guess you could say, some sense of comfort or hope to get me through it.” [Non-Hispanic, Black, 35 years, female, 6 children] “I feel like breastfeeding was the one thing that I was shocked by how hard it was. So, getting more support and encouragement would have been nice ... Feel like it’s not just newborn care. That’s also postpartum. My body too.” [Non-Hispanic, White, 30 years, female, 2 children]
Chatbot usability	“I think moms are getting the most information from their phones, and kind of a digestible bite size thing when you can, like, occasionally glance at your phone for a few minutes is helpful.” [Hispanic/Latina, Other, 23 years, female, 2 children] “It was really easy. I don’t have any real complaints about it. It was very simple to use, in my opinion.” [Non-Hispanic, Black, 23 years, female, 2 children] “I had the information through text and email, and just the resources were really helpful, and then just how quickly the chatbot worked. And just that it also felt like I was talking to a real person, it felt like a very sophisticated robot ... And I remember, like, sharing those resources with my husband, too. So it was nice, because it like also added a source of credibility.” [Non-Hispanic, White, 31 years, female, 1 child] “Overall, just very helpful. And I thought it was really amazing to see ... just considering the generation that we are in, tech is constantly evolving. And I feel like the world should start evolving more, especially medical wise, just with technology and adapting, certain people that are getting more into technology. So, I thought that was very refreshing. Like I said, it’s a very nice experience. Honestly, took me by surprise, I’m like, ‘Woah, hospitals are doing this now?’ Like this is great. But yeah, I thought that was pretty cool, too.” [Hispanic/Latina, Other, 19 years, female, 1 child]
Chatbot suggestions	“It comes in English and I have to put it in the translator, and it gets a bit more difficult.” [Hispanic/Latina, Other, 29 years, female, 3 children] “...If there’s some sort of way you can write in a question ... And even if it’s not something that the chatbot can respond to, it can say, we received your question and someone will reach out to you.” [Non-Hispanic, White, 30 years, female, 1 child] “So maybe an opportunity of like, send this to like your husband, or whoever, like they need support. I know I needed a lot of support in those first two weeks because you’re not supposed to go up and down the stairs. Things like that and how they can be supportive to you and the baby.” [Non-Hispanic, Black, 26 years, female, 1 child] “It would be great if all information appeared in one site that I could refer back to later and also have a choice of selecting which topic I want to learn about at any given time rather than a pre-determined order.” [Non-Hispanic, Asian, 37 years, female, 1 child—Survey] “...There could be a 12-week one, just because that’s when a lot of transitions and a lot of people are going back to work or things like that.” [Non-Hispanic, White, 30 years, female, 2 children]

Most participants in the survey (n=80, 80%) and interview liked the timing and frequency of the outreach messages, although a few survey participants (n=10, 10%) indicated that the messages were too frequent. Participants in the interviews and survey also recommended extending messaging to at least 12 weeks to support longer-term recovery and life transitions such as going back to work. One participant reported that she felt that the chatbot messages ended abruptly and recommended including a final message indicating that the chatbot experience was complete. Of note, one interview participant who opted out from the chatbot indicated regretting it and recommended including a summary of all the topics that will be covered before someone can opt out.

### Chatbot Content Usefulness and Recommendations

Most of the participants in the survey and interview found all topics included in the chatbots to be useful. Participants noted that the chatbot information complemented and reinforced the information provided by their providers and at discharge. Some of the participants spoke about how the content supported them through their journey in their recovery and caring for their newborn. Specifically, topics rated as useful by the greatest percentage of survey participants included appointment reminders (n=59, 59% for newborn appointments and n=66, 66% for postpartum appointments; full results in Table S2 in Multimedia Appendix 1), and nearly a quarter of participants (n=22, 22%) thought that the resources to schedule appointments were useful. Participants further highlighted the importance of receiving tailored information for postpartum recovery. One interviewee noted that the information was beneficial for sharing newborn recommendations with other caregivers.

For the newborn chatbot, 86% (n=86) of the participants found the warning sign information to be useful and 58% (n=58) of the participants found the parental leave information to be useful. For the postpartum chatbot, 88% (n=88) and 86% (n=86) of the participants found the warning signs and postpartum depression information to be useful, respectively (Multimedia Appendix 1). For both the newborn and postpartum chatbots, interview participants recommended more information about breastfeeding (eg, engorgement or mastitis, how to latch, access to a pump, resources for lactation consultants, and differences between breast milk vs formula) and parental leave. Most participants also recommended that the resources to schedule and attend medical appointments (eg, transportation, childcare, or insurance recommendations) should be offered to all participants regardless of whether they indicate a need. Participants also requested information on how to improve communication with providers and more resources for emotional and practical support.

For the postpartum chatbot, survey and interview participants recommended adding additional information about postpartum recovery including mental health warning signs and support, vaginal birth, pain management, pelvic floor issues, and monitoring blood pressure. For the newborn chatbot, participants noted that it was important to add additional information about what is normal versus concerning for a newborn (eg, number of wet diapers, bowel movement texture and color, baby acne, cradle cap, umbilical cord, and circumcision recovery). Some participants also recommended adding specific tips on newborn care, such as changing newborn diapers, newborn sleep recommendations (eg, number of recommended hours, wake windows, and establishing sleep routines), and activities to do with a newborn. Participants with specific needs recommended additional targeted information; for example, participants with a premature newborn recommended adding specific information about premature newborns (eg, expected weight gain and recommended room temperature), participants with twins recommended including practical recommendations on how to care for twins, and participants with older children suggested more information about how to care for a newborn with older siblings at home.

### Perceived Ease of Use

The majority of survey (n=81, 81%; Table 4) and interview participants who engaged with the chatbot found the chatbot easy to use. During the interviews, participants described the chatbot as personable and practical. Participants also liked that they could use their smartphones to open the chatbot. Most of the participants in the survey (n=72, 72%) and interviews indicated that they would recommend the chatbot to other people. Participants in the survey and interviews provided different suggestions to improve the delivery of chatbot information, which included a video or voiceover option, an option for patients to provide immediate feedback after navigating each experience, an option to share the chatbot content with their partner or other caregiver, and an option to easily go back to information of interest. Several participants recommended adding the capability to interact with a provider via the chatbot to answer nonurgent questions. Finally, Spanish-speaking participants recommended having the chatbot available in Spanish.

## Discussion

### Principal Findings

We found that the chatbot is a promising strategy to deliver relevant and timely education and referral to resources for birthing and caregiving individuals. To our knowledge, this is the first report on perceptions of a postpartum or newborn chatbot delivered at scale as part of usual care upon hospital discharge.

Previous studies illustrated preliminary interest in chatbots as a modality to receive information about pregnancy and postpartum concerns. Researchers who developed an artificial intelligence-based, open-ended question-and-answer chatbot using a community-engaged approach with pregnant women and new mothers of color also conducted community demonstrations to solicit feedback. They queried participants on interest in using a chatbot to receive information and found that 109 participants were highly receptive [20]. Our findings further expanded on the previous study by evaluating outreach strategies on how to best reach and engage patients by email and SMS text message.

This study suggests that the chatbot provided useful and timely information during the postpartum period. This is particularly relevant, as in a study with Black new mothers, none of the participants were able to identify more than five of nine key postpartum warning signs [29]. While we were unable to conduct a pre-post knowledge test through the chatbot, interview participants reported limited knowledge of key topics prior to discharge and found the postpartum warning signs and mental health information to be useful. Similar outreach and chatbot strategies targeting postpartum mental health issues have improved birthing individuals’ well-being [23,24,30]. One study targeting postpartum depression found that patients who engaged highly with an AI chatbot had lower symptoms of depression during the postpartum period compared to patients with low engagement [23]. Other studies found that engaging with an SMS text-messaging program can reduce postpartum anxiety [24], and web-based courses that provide information to birthing individuals can lower anxiety and result in fewer emergency visits compared to standard of care [30]. Nevertheless, additional research is needed to understand how chatbots can serve to fill knowledge gaps in postpartum health care and impact postpartum and newborn health outcomes.

Chatbots provide a potential opportunity to create a sense of support for new parents or birthing individuals, which is important since social support is identified as a protective factor for postpartum depression and anxiety [31]. Our finding that more than 10% of the participants in the surveys and interviews reported that they did not have support for themselves or taking care of their baby after hospital discharge suggests an avenue for at least informational support through consistent outreach. Content also included in-person and web-based support communities that might be able to fill some of the gaps in support after delivery.

This study also elucidated postpartum individuals’ and newborn caregivers’ health information needs about postpartum and newborn care after discharge from the hospital. In general, participants desired more information about breastfeeding, postpartum recovery, mental health, and newborn care among other content areas. Participants who were planning to go back to work wanted more information about parental leave. These findings are similar to a scoping review reporting that nurses prioritized safety and avoiding adverse events after discharge (eg, the prioritized topics in our chatbot) and patients prioritized self-care, pain management, infant care, and parenting (eg, the topics many participants wanted to hear more about) [32]. Thus, increasing engagement with the newborn and postpartum chatbots may require including more material desired by patients to also deliver information about warning signs.

### Limitations and Future Directions

While this study had an adequate representation of individuals from historically ethnically or racially minoritized populations, the chatbot was delivered in English and data collection was completed in English and Spanish. Although we shared screenshots of the outreach messages, chatbots, and topics covered by the chatbots during the interviews, the survey and interview relied on participants’ recall of their experiences with the chatbot, which may have gotten confused with other outreach strategies they received including messages related to apps from their prenatal providers such as Babyscripts. Similarly, study participants might have more positive experiences with the chatbot compared to those we did not survey or interview. We attempted to mitigate this by including both people who did and did not engage with the chatbot. Finally, though participants indicated that the information provided in the chatbot was useful, we did not evaluate the impact of the chatbot on participants’ knowledge, access to care, and overall well-being. Future research to examine how chatbot engagement affects patient knowledge about warning signs, appointment attendance, and other patient health outcomes is needed.

### Conclusions

The postpartum and newborn chatbots were identified as an acceptable and useful strategy to provide information and resources to birthing individuals and newborn caregivers about postpartum recovery and newborn care. Nevertheless, future work should measure the impact of chatbots on specific postpartum and newborn health outcomes including health disparities.

## Appendix

Multimedia Appendix 1 Additional tables.

## Abbreviations

AI: Artificial Intelligence
EHR: electronic health record
HIPAA: Health Insurance Portability and Accountability Act
REDCap: Research Electronic Data Capture

## Data Availability

The datasets generated during or analyzed during this study are available from the corresponding author upon reasonable request.
