# Supplementary material for: Development and Refinement of a Chatbot for Birthing Individuals and Newborn Caregivers: Mixed Methods Study
Source: JMIR Pediatr Parent. 2024 Nov 14;7:e56807. doi: 10.2196/56807 (PMC11605260; doi:10.2196/56807)
Supplement: Multimedia Appendix 1 [file pediatrics_v7i1e56807_app1.docx]

**Table S1.** Newborn and postpartum chatbot content by day of outreach message.

| Outreach postdischarge day | Newborn topic | Postpartum topic | Example of resources |
| --- | --- | --- | --- |
| Day 1^a^ | - Pediatric appointment reminder - Resources to address common challenges for scheduling the newborn appointment - Parental leave | - Postpartum warning signs - Baby blues | - Pediatrician contact information - District Direct App - Healthy Children - DC Healthy Families - March of Dimes |
| Day 3 | N/A^b^ | - Postpartum warning signs - C-section recovery tips - Postpartum visit - Resources to address common challenges for scheduling the postpartum appointment | - Association of Women’s Health, Obstetric and Neonatal Nurses - Providers contact information |
| Day 7 | - Pediatric warning signs - Newborn sleep recommendation - 1 month appointment reminder | - Maternal nutrition | - National Institute of Health Safe Sleep Environment - American Pregnancy Association-Breastfeeding nutrition |
| Day 14 | - Newborn nutrition recommendations - Resources for breastfeeding and formula - Food assistance program | - Postpartum depression resources - Sleep recommendations | - CDC^c^: breastfeeding and infant formula information - The Special Supplemental Nutrition Program for Women, Infants, and Children - DC Breastfeeding Coalition - Postpartum Support International - Maternal Mental Health Hotline |
| Day 21 | N/A | - Sex after birth - Family planning | - Mayo Clinic: sex after pregnancy - March of Dimes |
| Day 28 | - Newborn developmental milestones | - Kegel exercises | - Small Moment, Big Impact - Mayo Clinic: Kegel exercises |
| Day 38 for newborn or  Day 42 for postpartum | - Recommended pediatric visits and vaccines | - Social support - Postpartum depression reminders | - CDC: recommended vaccine schedule - DC Metro Perinatal Mental Health - National Suicide Prevention Hotline |

^a^Caregivers received up to 3 daily newborn follow-up reminders until the caregiver indicated that the newborn appointment was scheduled.

^b^Not applicable.

^c^CDC: Centers for Disease Control and Prevention.

**Table S2.** Chatbot content feedback from the survey (n=100)

| Newborn topic usefulness | n |
| --- | --- |
| **Visit reminders** |  |
| Strongly agree/agree | 59 |
| Neutral | 26 |
| Strongly disagree/disagree | 9 |
| Not applicable | 6 |
| **Warning signs** |  |
| Strongly agree/agree | 86 |
| Neutral | 9 |
| Strongly disagree/disagree | 2 |
| Not applicable | 3 |
| **Sleep** |  |
| Strongly agree/agree | 73 |
| Neutral | 17 |
| Strongly disagree/disagree | 4 |
| Not applicable | 0 |
| **Feeding** |  |
| Strongly agree/agree | 76 |
| Neutral | 18 |
| Strongly disagree/disagree | 2 |
| Not applicable | 4 |
| **Milestones** |  |
| Strongly agree/agree | 74 |
| Neutral | 14 |
| Strongly disagree/disagree | 1 |
| Not applicable | 11 |
| **Vaccinations** |  |
| Strongly agree/agree | 73 |
| Neutral | 16 |
| Strongly disagree/disagree | 1 |
| Not applicable | 10 |
| **Parental leave** |  |
| Strongly agree/agree | 58 |
| Neutral | 19 |
| Strongly disagree/disagree | 4 |
| Not applicable | 19 |
|  |  |
| **Postpartum topic usefulness** |  |
| **Visit reminders** |  |
| Strongly agree/agree | 66 |
| Neutral | 17 |
| Strongly disagree/disagree | 9 |
| Not applicable | 8 |
| **Warning signs** |  |
| Strongly agree/agree | 88 |
| Neutral | 9 |
| Strongly disagree/disagree | 1 |
| Not applicable | 2 |
| **Sleep** |  |
| Strongly agree/agree | 69 |
| Neutral | 17 |
| Strongly disagree/disagree | 2 |
| Not applicable | 12 |
| **Nutrition** |  |
| Strongly agree/agree | 67 |
| Neutral | 21 |
| Strongly disagree/disagree | 1 |
| Not applicable | 11 |
| **Postpartum depression** |  |
| Strongly agree/agree | 86 |
| Neutral | 8 |
| Strongly disagree/disagree | 1 |
| Not applicable | 5 |
| **Family planning and sex after birth** |  |
| Strongly agree/agree | 72 |
| Neutral | 17 |
| Strongly disagree/disagree | 3 |
| Not applicable | 8 |
| **Kegel exercises** |  |
| Strongly agree/agree | 65 |
| Neutral | 15 |
| Strongly disagree/disagree | 6 |
| Not applicable | 14 |
| **Social support** |  |
| Strongly agree/agree | 65 |
| Neutral | 22 |
| Strongly disagree/disagree | 5 |
| Not applicable | 8 |
